# Clinical psychopathology-based early relapse prediction model using speech and language in psychosis

**DOI:** 10.1016/j.scog.2025.100392

**Published:** 2025-09-23

**Authors:** Tyler C. Dalal, Min Tae M. Park, Angelica M. Silva, Svetlana Iskhakova, Alban Voppel, Noah J. Brierley, Michael MacKinley, Emmanuel Olarewaju, Lena Palaniyappan

**Affiliations:** a Schulich School of Medicine and Dentistry, Western University, London, ON, Canada; b Douglas Mental Health University Institute, Department of Psychiatry, McGill University, Montreal, QC, Canada; c Department of Psychiatry, Western University, London, ON, Canada; d Robarts Research Institute, London, ON, Canada; e Department of Psychology, Laurentian University, Sudbury, ON, Canada; f Writing-brain Laboratory, Brandon University, Brandon, MB, Canada

**Keywords:** Disorganization, Impoverishment, Thought disorder, Computational linguistics, Precision psychiatry, Relapse, Early intervention

## Abstract

**Introduction:**

Prediction of psychotic relapse using speech-derived markers promises targeted early intervention. However, the sheer number of speech markers and the ‘black box’ nature of predictive models challenges clinical translation.

**Methods:**

We propose a psychopathology-based systematic approach to identify likely relapse. We draw on the notion that the predictors of relapse should mark (1) the presence of schizophrenia in its untreated early stages and (2) track disorganization in psychosis. By leveraging Natural Language Processing, we derive 3 lexical, syntactic and narrative markers -semantic similarity, clause complexity, and analytic thinking index from speech samples of people with acute psychosis (*n* = 68) followed up for subsequent relapses over a year (12 out of 68).

**Results:**

Speech-based model predicted relapse status with strong evidence (Bayes Factor BF_10_ = 79.5) against the clinical intuition model.

**Conclusion:**

Using a Bayesian approach, this preliminary study demonstrates the utility of psychopathology-guided variable selection for speech-based relapse prediction complementing clinical intuition in practice.

## Highlights

• Speech markers may have greater predictive utility for relapses than clinical intuition.
• Analytic thinking and clause complexity relate to relapse risk.
• Positive thought disorder severity relates to early relapse.
• Brief speech samples provide prognostic information.
• Psychopathology-guided feature selection enhances model interpretability.

## 1 Introduction

Automated speech markers recovered using Natural Language Processing (NLP) methods, including corpus linguistic approaches, are increasingly employed in a predictive analysis framework (Corona Hernández et al., 2023). Several studies have demonstrated the feasibility of using NLP markers in predictive models for psychosis-related outcomes (Dalal et al., 2024; Tan et al., 2021; de Boer et al., 2020b; Corcoran and Cecchi, 2020). Indeed, there is emerging evidence for differentiating schizophrenia from other psychotic disorders (Mota et al., 2017; Tan et al., 2021) and in predicting the onset of the first episode (Corcoran et al., 2018; Spencer et al., 2021). A third potential emerging application is in predicting the second episode, or more broadly, relapses and readmissions in psychosis (Zaher et al., 2024).

Clinical prediction models that aim to provide individual-level information are critical tools for precision psychiatry, but two major inter-related obstacles exist in bringing them to routine clinical use. One is the ‘black box’ nature of predictions (Rudin, 2019; Salazar de Pablo et al., 2021), which often makes it difficult for human users to know why a prediction works or fails in the clinical setting where observations are made. Second is the large feature space derived from numerous automated measures, giving rise to a combinatorial explosion that reduces replicability and successful external validation. This issue, termed *the curse of dimensionality*, is particularly relevant for speech markers where multiple domains of information can be extracted as variables (Berisha et al., 2021).

Huys et al. (2016) advocates for using data reduction methods (e.g., principal component analysis), penalizing complexity (e.g., via regularization approaches or Bayesian Model Selection) or employing a theory-driven approach to extract meaningful low level parameters representing the processes that generate the high dimensional data. Unlike the first two data-driven solutions, the theory-driven approach can improve interpretability as well as reduce dimensionality (Rudin, 2019). Nevertheless, one of the major challenges in theory-driven approaches at the forefront of computational psychiatry is that the biophysical/neurobiological process theories do not directly model clinical outcomes such as relapses, but usually address certain constrained task performance (e.g., reinforcement learning, decision making). As a result, despite the mechanistic clarity they offer, they remain unusable for clinicians (Hauser et al., 2022).

**Fig. 1:**
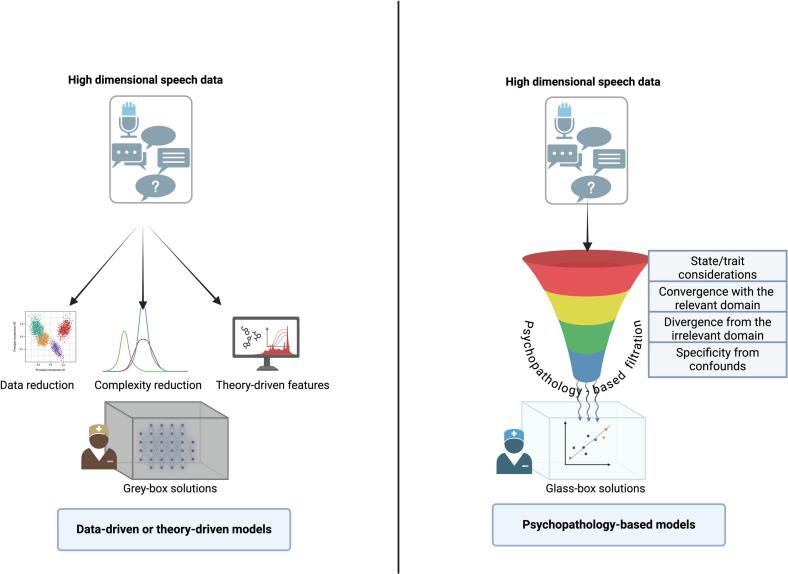
Psychopathology-based prediction of clinical outcomes using speech data. Left: Computational psychiatry aims to predict clinical outcomes using a data-driven or theory-driven framework. When faced with high-dimensional data with multiple features, data reduction can occur at preprocessing phase (data reduction), or during predictive model building (complexity reduction). Alternatively, mechanistic theories (e.g., drift-diffusion model of evidence accumulation) can provide low dimensional parameters that capture the underlying biophysical or cognitive processes. Even for successful data/theory-driven predictions, clinical sense-making remains a challenge (black or greybox solutions). Right: Psychopathology-based models of clinical outcomes take a top-down approach. We first start with identifying the psychopathological phenomenon that is relevant for the outcome of interest. For example, to model the outcome of future psychotic relapses at the first presentation, the selected speech-NLP features must be more prominent during the initial first episode state (state/trait effect), vary with positive but not negative FTD or explained by medications (causal symptom). This filtration helps to first identify the features that are most likely to relate to the psychopathological phenomena, followed by model evaluation. The resulting predictions are more likely to be clinically accessible, as they follow what a clinician expects to see with the outcome of interest. Of note, the selection process can begin as a data-driven search (e.g., check the NLP feature that best correlates with the psychopathological features of interest, showing periodicity, associated with positive FTD, dissociated from negative FTD and not affected by medication confounds, in the case of psychotic relapses).

In computational psychiatry, the theory-driven approaches have mostly been based on biophysical models (e.g. microcircuit and hemodynamic models of fMRI timeseries; (Rajapakse et al., 1998)) or cognitive models (e.g. reinforcement learning, active inference; (Montagnese et al., 2020; Barch et al., 2017)) of data generation. But such formalized physiological explanations of the data generation processes are lacking for many ethological behavioral paradigms, including speech (but see Sharpe et al., 2025; Wang et al., 2025). In these instances, we argue that psychopathological theory (i.e. empirically validated prior knowledge on the psychopathological basis of both the outcome and the predictors) can provide a means to select the relevant features to build a transparent and interpretable prediction model. In essence, this introduces clinical theory into precision psychiatry. In practice, this *filtration* can be done sequentially using Bayesian statistical approaches, by first identifying the psychopathological construct that is most related to the outcome (*construct identification*); then selecting automated markers from a constrained set that converge on the chosen construct but diverge from unrelated constructs (*variable selection*); and finally, a Bayesian Model Selection to compare the ‘derived model’ based on predictors built from theoretical filtration against ‘external comparator models’ without automatic/ethological predictors (Fig. 1). The external models could be based on clinical intuition (e.g., a cannabis-using, poorly functioning male patient is more likely to relapse).

*Psychopathology-based models* are not ‘process models’, but are best seen as early-stage ‘working models’ that offer *constitutive* (i.e., how can a model [e.g., a set of speech-based variables] make an outcome [e.g., relapse] prediction work?) rather than *causal* explanations (i.e. what leads to the outcome e.g., the temporal or mechanistic antecedents such as the idea that dopamine dysregulation causes relapse; (Kind and Dayan, 2024)). For such models, initial considerations involve the clinical psychopathology of what constitutes the outcome and the predictors. We employ ‘relapse prediction’ as an exemplar case that maps on disorganization as a state-related psychopathological construct, but the approach proposed here can be used for many relevant clinical outcomes (e.g., treatment response, depressive episodes, suicidal crisis, employment success etc.).

### 1.1 Psychopathology of outcome (relapse)

A psychotic relapse is an accentuation of the positive symptoms on which the initial diagnosis is often based (Emsley et al., 2013; Olivares et al., 2013). It is psychopathologically distinct from other help-seeking events that occur in the course of a psychotic disorder (e.g., depressive relapse, situational crisis, exacerbation of negative symptoms (Häfner, 2019). Thus, markers that predict psychotic relapses can be expected to relate to the construct of an *initial untreated symptomatic state* (first-episode), which, in the same psychopathological form, repeats itself as relapses. The speech markers relevant for psychosis are presumed to result from two distinct latent psychopathology: disorganization (or positive formal thought disorder (FTD) and impoverishment of thinking (or negative FTD). Negative FTD is a stable trait-like feature that may have specific value in detecting the emergence of the clinical profile of a long-term psychotic disorder such as schizophrenia. Many recent studies have indicated that negative FTD related speech features predict the onset of psychosis over 2 years (Spencer et al., 2021; Agurto et al., 2023; Bedi et al., 2015), but evidence predicting subsequent episodes (relapses) are less clear-cut. While some studies link negative FTD with relapse and re-hospitalization (Wilcox et al., 2012; Wilcox, 1990), (Bechdolf et al., 2002), most others find positive FTD as more indicative of relapse (Harrow and Marengo, 1986; Jørgensen and Aagaard, 1988; Roche et al., 2016; Bouhlel et al., 2012). This discrepancy may arise from FTD being assessed after several months of antipsychotic treatment in many studies. Unlike the ‘trait-like’ negative FTD, the state-like positive FTD reduces in severity over the first few months of psychosis (Ucok et al., 2021), partly due to its responsiveness to antipsychotics (Spohn et al., 1986); thus its predictive value is best estimated before long-term treatment exposure.

### 1.2 Psychopathology of predictors (speech features)

Several linguistic markers (i.e. speech-derived, language-based but not voice-related, Natural Language Processing or speech-NLP markers) differ in persons with psychosis compared to those without (Dalal et al., 2024; Ehlen et al., 2023), but only a few have been studied in the first-episode state as opposed to prodromal stages or chronic stages. In this regard, three automated linguistic markers are of special interest: 1. Semantic similarity or coherence (i.e., choosing words with similar meaning representation to each other when speaking and may reflect repetitive word use) (Alonso-Sánchez et al., 2022) 2. Analytic Thinking Index (Pennebaker et al., 2014) (a metric that differentiates a more formal, structured communication from a less formal, more narrative style based on function words such as articles and prepositions, suggesting disorganized thought structure) (Silva et al., 2021) and 3. Clause complexity (i.e., the grammatical structure of clauses used in speech production and which a low clause complexity suggests grammatically simple sentences) (Silva et al., 2023). These three indices capture the 3 crucial levels of language dysfunction (word-level choices, grammar and discourse style), all of which are suspected to be affected by the pathophysiology of schizophrenia (Hinzen and Palaniyappan, 2024; Elleuch et al., 2025; Kuperberg, 2010). We use these 3 variables for the proof-of-concept work presented here.

Speech-derived NLP markers with clinical promise in psychosis tend to track disorganization and/or impoverishment, supporting their construct validity (Bilgrami et al., 2022). Higher semantic similarity tracks negative aspects of FTD (Alonso-Sánchez et al., 2022), while Analytic Thinking Index varies with the severity of positive FTD (Silva et al., 2021; Limongi et al., 2022). Clause complexity, on the other hand, has a notable overall diagnostic effect in schizophrenia (Silva et al., 2023; Schneider et al., 2023; Elleuch et al., 2025) and appears to relate to both positive (Çokal et al., 2018) and negative FTD features (Ciampelli et al., 2023; Jeong et al., 2023).

In the current study, we assess the relapse prediction potential of automated markers obtained from an active 3-minute speech sample from a picture description task during an untreated acutely psychotic state in first-episode patients. This cohort is of particular interest as patients had only 2 median days of defined dose exposure when the speech sample was obtained, reducing the confound due to the long-term effects of hospitalization (King et al., 1990; Thomas et al., 1990) and antipsychotic exposure that can affect language functioning (de Boer et al., 2020a). Secondly, following clinical remission from acute psychotic symptoms (i.e., achieving CGI < 3 and > 50 % reduction in core symptom burden PANSS-8, as reported in a subsample in (Dempster et al., 2020), we followed up these patients for 1 year by tracking every hospital admission (both in emergency and psychiatric inpatient settings, if >24 h) due to worsening psychotic symptoms, enabling a prospective verification of the construct validity of the NLP variables. Our findings provide evidence of incremental value for automated speech analysis in early relapse prediction in the first year of treating psychosis.

## 2 Methods

### 2.1 Participant recruitment

73 English-speaking individuals with first-episode psychosis (FEP) from an ongoing project, Tracking Outcomes in Psychosis (TOPSY; ClinicalTrials.gov Identifier: NCT02882204), were included in this study. The FEP participants included in this study are part of a larger ongoing longitudinal study that includes FEP, schizophrenia, and chronic-high risk individuals. Patients with FEP were recruited from Prevention and Early Intervention for Psychosis Program (PEPP) at the London Health Sciences Centre in London, Ontario which is an early intervention program. Patients were recruited between 2017 and 2020 upon first presentation and FEP participants were assessed within first week of referral to the program. Exclusion criteria were chosen with the purpose of reducing confounders and included a history of head injury, intellectual disability or other medical conditions (e.g. untreated hypertension, diabetes, hepatic/rental insufficiency, neurological condition), a history of drug or alcohol dependence with the past year or being unable to provide informed consent. All participants provided written informed consent as per the Western University Health Research Ethics Board approval.

### 2.2 Assessments

**Fig. 2:**
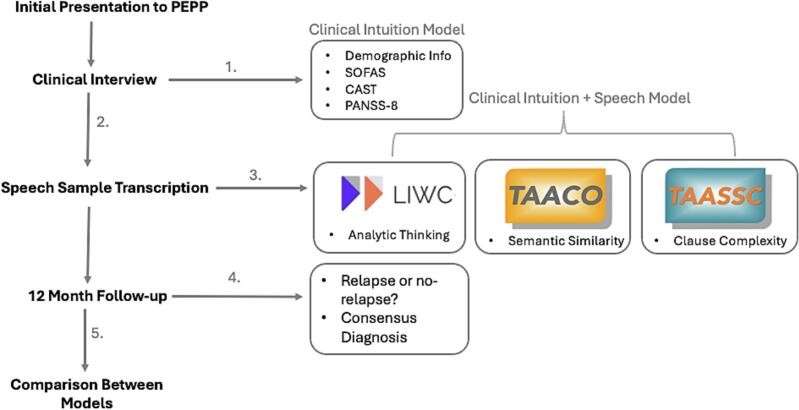
Visual representation of the methodology. Patients referred to the first episode psychosis clinic (PEPP, London, Ontario) from various sources in the community (self-referral, social worker, family doctors or other physicians). 1) Clinical interviews were conducted in the first week of presentation. 2) Speech samples were obtained using the Thought and Language Index procedures. 3) Speech data transcribed and analyzed by three different language processing tools. 4) A 12-month retrospective chart review after program entry and clinical stabilization is conducted to determine whether the patient relapsed (hospitalization within the next year), and a consensus diagnosis is confirmed. 5) Patients were grouped based on relapse or no-relapse; speech and clinical variables were collected at baseline and then correlated with the later relapse status.

Subjects were assessed upon first presentation using the Positive and Negative Syndrome Scale-8 (PANSS-8 (Lin et al., 2018)), the Social and Occupational Functioning Assessment Scale (SOFAS, operationalized in line with (Morosini et al., 2000)), and the Cannabis Abuse Screening Test total score (CAST (Legleye et al., 2015)) indicating the severity of self-endorsed cannabis use. We summarized scores on two subscales from PANSS-8 (positive symptoms and negative symptoms). Baseline antipsychotic exposure was quantified from the World Health Organization's Daily Defined Dose (DDD; (WHO Collaborating Centre for Drug Statistics and Methodology, 2003) which provides a common unit of exposure to antipsychotics. Speech was assessed using 3 picture prompts as part of the procedures for the Thought and Language Index (TLI; (Liddle et al., 2002)), which is an interview-based instrument to assess 2 types of formal thought disorder (poverty of speech and weakening of goal summed up as *TLI*-*impoverishment*; looseness, peculiar word, sentences and logic summed up as *TLI-disorganization*). The clinical intuition model was based on commonly accepted demographic (age, education, cannabis use severity) and clinical features (SOFAS) which was collected at first contact. These variables represent a common heuristic utilized by clinicians to anticipate relapse (i.e., cannabis-using, low-functioning males are more likely to relapse) based on the information available at first presentation. The status of relapse at one year was determined via retrospective chart review by two independent psychiatrists (MTMP, SI); we had *n* = 12 relapses. As speech-based predictors, we included markers at the lexical (Analytic Thinking), syntactic (Clause Complexity), and semantic (Semantic Similarity) levels (Fig. 2). More details on the relapse criteria and details of language variables are provided in Supplemental materials.

### 2.3 Statistical analysis

To demonstrate the feasibility and the utility of psychopathological construct-guided predictive modelling in this field, we leverage a stepwise, 3-stage Bayesian analysis (Step 1 - *construct identification*, step 2 - *variable selection* and 2 - *model evaluation*). This involves selecting the target psychopathological domain with higher severity among those who later relapsed. Then, we assess the inclusion probabilities of selected NLP variables based on their convergence with the chosen FTD domain and divergence from the opposite domain. Finally, we conducted Bayesian model selection procedures to assess the evidence for speech-based relapse prediction, first as a stand-alone model, then in combination with a clinical intuition model of relapse based on the information available to clinicians at first presentation.

All statistical analyses were conducted using Bayesian approaches via JASP (Love et al., 2019). In step 1, *construct identification,* we use two independent-samples *t-*tests using comparing TLI disorganization and impoverishment scores between relapse and non-relapse groups to narrow down the variable search, requiring at least moderate evidence (Bayes Factor; BF10) >3 for inclusion. In step 2 (*variable selection*), we conducted two separate Bayesian Linear Regression analyses with the same set of three speech-NLP variables as predictors for disorganization and impoverishment to identify NLP variables with Bayes Factors (BF10) >20. for inclusion or exclusion in the relapse prediction model, depending on the TLI domain to which they relate. To balance overfitting and loss of information, factors with strong evidence of relationship with the FTD domain that did not differ between relapse/non-relapse were excluded, while those with equivocal evidence were retained. In step 3 (*model evaluation* for relapse prediction), we compared an intuitive clinical model of relapse-proneness (a low-functioning man with poor premorbid achievements and current active cannabis use) against a model defined by clinical and speech variables. For all regression analyses, we applied a beta (a = b = 1) prior to the models, which assumes that model sizes are equally likely a priori (JZS prior with JASP's default of *r* = 0.354 used). We report model-averaged inclusion probabilities of the predictors.

## 3 Results

### 3.1 Construct identification

**Fig. 3:**
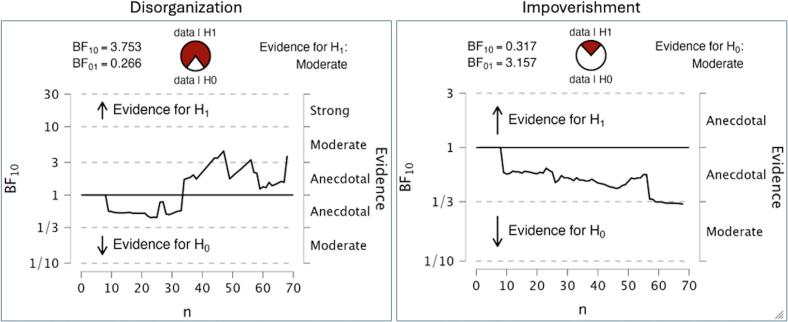
Sequential analysis of construct identification. JASP output of sequential analysis shows the evidential flow in favour of H1 for disorganization scores (left) from the Thought and Language Index (TLI) being different (higher) among those who showed an early relapse later vs. those who did not. The evidence favours a lack of difference between the relapse/non-relapse groups for impoverishment scores (right). Each point indicates a change in the BF when a participant is added in the analysis (n in x axis). BF = Bayesian Factor. H_0_ - null hypothesis (of no difference). H_1_ - alternative hypothesis.

We first sought evidence for the rating scale scores of TLI disorganization in relapse prediction, by testing for association between TLI scores and later relapses. TLI disorganization index received a BF10 > 3.75 indicating a moderate degree of support for the hypothesis of positive FTD being associated with future relapse (mean(sd) = 0.566(0.648) in non-relapse; 1.198 (1.265) in relapse group). TLI impoverishment received BF10 of 0.32, with moderate evidence in favour of the null hypothesis i.e., there is no difference in negative FTD between the two groups (mean(sd) = 0.396(0.473) in non-relapse; 0.361 (0.492) in relapse group). This result (Fig. 3) supports the selection of positive FTD related variables related to TLI Disorganization as the domain of interest and exclusion of negative FTD for building a relapse prediction model in our sample.

### 3.2 Variable selection

We then conducted two linear regression analyses, one each examining if TLI disorganization and impoverishment are predicted by the NLP variables - Semantic Similarity, Analytic Thinking Index and Clause Complexity. The model with Analytic Thinking Index explained 27 % of variance (R^2^) in TLI Disorganization with very strong evidence against the intercept-only null model (BF_10_ = 2735). Analytic Thinking Index had strong evidence for inclusion (coefficient b(sd) = −1.354 (0.299);BF_inclusion_ = 23.39) while both Semantic Similarity (b(sd) = 0.082 (0.388);BF_inclusion_ = 0.385) and Clause Complexity (b(sd) = −0.022(0.080);BF_inclusion_ = 0.405) had weak evidence for being excluded from the models predicting positive FTD. The model with all three NLP variables explained 22 % of variance (R^2^) in TLI Impoverishment with strong evidence against the null model (BF_10_ = 111.8). Semantic Similarity had strong evidence for inclusion (coefficient b(sd) = −1.206 (0.48);BF_inclusion_ = 23.399) while both Analytic Thinking Index (b(sd) = 0.257 (0.228);BF_inclusion_ = 2.551) and Clause Complexity (b(sd) = 0.065(0.083);BF_inclusion_ = 1.454) had weak evidence for inclusion in the models predicting negative FTD.

In summary, our search for NLP variables for building the relapse prediction model favoured the inclusion of Analytic Thinking Index based on its relationship with TLI Disorganization and exclusion of Semantic Similarity based on its relationship with TLI Impoverishment. At this stage, Clause Complexity had insufficient evidence for exclusion from further analysis.

### 3.3 Relapse prediction model evaluation

Based on the above results, we chose TLI Disorganization, Analytic Thinking Index and Clause Complexity as speech-based predictors to be compared against the ‘clinical intuition model’ without information related to disorganized speech, but represented by known clinical/demographic markers of relapse available at the time of first clinical contact (Sex, years of education as a proxy measure of premorbid function, cannabis abuse screening test [CAST] score and Social and Occupational Functioning Score [SOFAS]). The speech-based model with TLI Disorganization and the two NLP variables explained 27 % of the variance (R^2^) in relapse status with strong evidence versus the clinical intuition model (BF_10_ = 79.476). Clause Complexity had the best evidence for inclusion (coefficient b(sd) = −0.181 (0.077);BF_inclusion_ = 23.399) while both TLI Disorganization (b(sd) = 0.085 (0.063); BF_inclusion_ = 5.898) and Analytic Thinking Index (b(sd) = −0.245(0.198);BF_inclusion_ = 4.817) had moderate evidence for inclusion in the models predicting relapse.

**Fig. 4:**
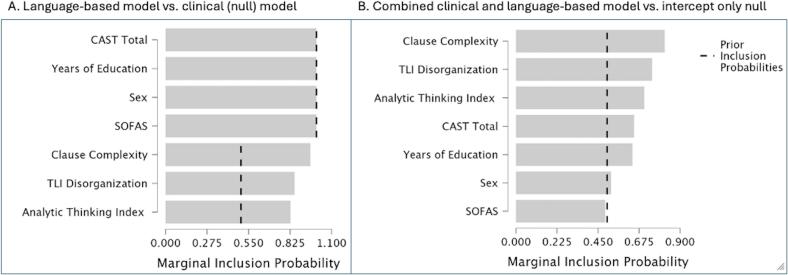
Model inclusion probabilities. JASP output of model inclusion probabilities for A) Language-based vs. clinical profile (cannabis-smoking man with premorbid educational deficits and low functioning) B) Combined language and clinical profile-based model v. clinical intuition (intercept only) model. Bars indicated model-averaged posterior inclusion probabilities for the Bayesian linear regression with dashed lines representing the prior probabilities (before the data is evaluated). CAST: Cannabis Abuse Screening Test score, SOFA: Social and Occupational Functioning Assessment Scale score, TLI: Thought and Language Index.

We also estimated the strength of a relapse prediction model that included all the chosen clinical-demographic and speech variables as predictors (simulating a clinician who has access to speech variables) to be compared against an intercept-only null model. There was strong evidence favoring the combination of all predictors in relapse prediction (R2 = 27 %, BF_10_ = 12.044). In this full model as well, Clause Complexity had the best evidence for inclusion (coefficient b(sd) = −0.146 (0.095); BF_inclusion_ = 4.419) while all other predictors (BF_inclusion_ = 1.088 to 2.951; TLI Disorganization >Analytic Thinking Index >CAST>Years of Education>Sex) had anecdotal evidence for inclusion when predicting relapse. SOFAS had weak evidence favoring exclusion (BF_inclusion_ = 0.959) from the relapse prediction models. All model inclusion probabilities are shown in Fig. 4. The distribution of individual predictors in the relapse and non-relapse groups is shown in Table 1.

**Table 1:** Distribution of demographic and clinical variables in Relapse and No-Relapse groups at the baseline.

Mean (standard deviation)	Relapse (*n* = 12)	No Relapse (*N* = 56)	Total sample (*N* = 68)
Gender (male/female)	9/3	46/10	55/13
Age	21.83 (4.93)	22.38 (4.32)	*P = 0.6974*
NS-SEC	2.67 (0.71)	3.50 (1.26)	*P* = 0.0313
Education years	15.00 (1.91)	14.56 (1.73)	*P* = 0.4351
Mean DDD	0.25 (0.25)	0.30 (0.51)	*P* = 0.7426
Diagnosis at follow-up	6 SZ, 1 PNOS, 1 SZA, 1 BP, 3 MDD	48 SZ, 4 PNOS, 2 SZA, 1 SZP, 1 BP	54 SZ, 5 PNOS, 3 SZA, 2 BP, 3 MDD, 1 SZP
Time to relapse (median)	28 days (IQR 22–90 days)	N/A	N/A
TLI Disorganization	1.20 (1.27)	0.57 (0.65)	*P* = 0.0144
TLI Impoverishment	0.36 (0.49)	0.40 (0.47)	*P* = 0.7914
SOFAS	41.67 (10.47)	41.02 (13.67)	*P* = 0.8774
CAST	9.17 (5.73)	11.30 (6.93)	*P* = 0.5810
PANSS-8 Total	25.45 (9.89)	25.12 (6.76)	*P* = 0.8886
PANSS-8 Positive	12.36 (4.13)	12.31 (2.97)	*P* = 0.9609
PANSS-8 Negative	8.27 (5.93)	7.44 (4.00)	*P* = 0.5535
ATI	0.26 (0.21)	0.44 (0.30)	*P* = 0.0528
Clause Complexity	6.74 (0.53)	7.03 (0.69)	*P* = 0.1757
Semantic Similarity	1.08 (0.12)	1.10 (0.12)	*P* = 0.6021

Notes: NS-SEC, national statistics socio-economic classification of parents. Education is classified as years in school (i.e. completed high school = 14; completed college = 16; completed university = 18). ATI = Analytic Thinking Index adjusted for word count; DDD = Defined Daily Dose of antipsychotics. SZA, Schizoaffective, SZP, Schizophreniform, BP, Bipolar, MDD, Major Depressive Disorder, PNOS, Psychosis Not Otherwise Specified. PANSS-8 Positive and Negative Symptom Scale, 8 item version.

## 4 Discussion

We expected speech-NLP variables that mark the early untreated phase of schizophrenia and are influenced by positive formal thought disorder to predict an early relapse (psychiatric hospitalization for positive psychotic symptoms after initial treatment response) over the first year of treatment. Using a series of Bayesian analyses in a sample of patients with untreated first-episode psychosis, we report 3 major observations: (1) The severity of positive FTD (disorganization) at the outset is higher in those who suffer an early relapse (2) Speech-based automated linguistic analysis of positive FTD strongly predicts future relapse, over and above a profile based on known clinical/demographic risk factors (3) Low clause complexity has a notable predictive value for relapse. Taken together, we provide preliminary proof for the concept that routine, non-invasive speech assessments that last 3 min can provide valuable prognostic information in first-episode psychosis.

We observed that positive but not negative FTD at the onset is an indicator of later relapse. It is important to note that our outcome of interest was the first relapse after the initial treatment response (i.e., reaching CGI < 3 in 4–12 weeks of treatment), and the relapses we observed occurred at a median interval of 28 days (IQR 22 to 90 days). Negative FTD may be more relevant for relapses that occur at a longer time scale (i.e., ‘late relapses’ (Wilcox et al., 2012; Wilcox, 1990) or the occurrence of more frequent relapses in a given individual. As we did not measure these variables, we cannot discount the role of negative FTD in predicting the risk of recurrent relapses over a longer timeframe.

Of the 3 NLP variables selected based on their relevance to the early phase of schizophrenia, we excluded semantic similarity and included the Analytic Thinking Index in the final model, given their observed relationships with the negative and positive FTD constructs, respectively. Such a clear construct-to-NLP-marker mapping was not possible for clause complexity, which was retained for assessment in the final model. In many prior studies, syntactic complexity has been measured using coarse measures of sentence or utterance length (Bilgrami et al., 2022; Liang et al., 2022). Length of these production units (sentences, utterances) drops when not much is spoken - a well-known issue even in the early phase of schizophrenia (Mackinley et al., 2023) - explaining their link with negative FTD (Bilgrami et al., 2022). Instead of these coarse measures of complexity, we used the more granular measure of clause complexity (Silva et al., 2023; Morice and McNicol, 1986), given its importance when sentence boundaries are unclear - a common issue for spoken language (Norris and Ortega, 2009). As clause complexity score is intrinsically controlled by the number of main clauses (and thus the amount of speech), its reduction is indicative of less complex thought, typical of ‘poverty of content’ in FTD scales (Liang et al., 2022). While poverty of content is conventionally seen as a negative FTD (Liddle et al., 2002; Roche et al., 2015; Stein et al., 2022; Zamperoni et al., 2024), some authors classify this with positive FTD (Kircher et al., 2014). A recent factor analysis of FTD ratings based on recordings with limited negative FTD burden reported poverty of content to be distinct from negative FTD (Tang et al., 2022). This unique positioning and sharing of the processes behind both positive and negative FTD might have afforded a predictive advantage to clause complexity over other variables. To our knowledge, the relationship between clause complexity and psychotic relapse reported here is a novel finding.

Currently, model building in precision psychiatry commonly involves using all possible variables in the feature space with the risk of overfitting or using automated approaches to select ‘top-n’ features. Both of these approaches bias the estimate of model performance (Meehan et al., 2022). While feature selection driven by clinical knowledge is seen as an important means to reduce the risk of bias in prediction models, a recent review points out that <9 % of 220 studies in the field of precision psychiatry use such theory-driven approaches (; Meehan et al., 2022). In part, the lack of use of clinical knowledge for predictive models is due to the uncertainty surrounding clinical reasoning and expectations. We show that the methodical use of Bayesian decision-making guidance can overcome this challenge, as in our case, where the inclusion of clause complexity in the tested model followed the lack of strong exclusionary evidence.

Our work is a proof-of-principle, albeit in a small sample, to demonstrate that psychopathology-based model building can improve our ability to predict relapse risk, over and above our clinical intuition. The speech-based model is based on 3-minutes of picture description – a test that can be easily performed during routine healthcare visits. Our results are consistent with several other groups reporting informative signals in speech to predict psychotic relapses (e.g., Crosscheck application: (Adler et al., 2020; Ben-Zeev et al., 2017; Buck et al., 2021; Lamichhane et al., 2023); Acoustic speech spectrogram using time-frequency architecture: (Garoufis et al., 2022; Zlatintsi et al., 2022)). Notably, some of these studies employ acoustic variables such as prosody, which are better suited as predictors of imminent relapse, especially if speech is acquired on a longitudinal basis (for a review, see (Zaher et al., 2024)). We anticipate the current advancements in AI-based transcription and computational analysis to soon enable speech variables to be parsed through in real-time, readily providing speech-based markers at the clinic.

The severity of positive formal thought disorder (FTD) and the presence of less complex and less structured speech expressions at the onset hold promise as prognostic indicators for early relapse. While the clinical theory behind our model is based on the relevance of disorganization/FTD to relapse, other clinical theories can also provide empirically testable predictions. For example, one may expect stress-induced speech changes to foretell imminent relapses; thus, one can select those NLP measures (e.g., sentiment analysis, worry-related lexicon) that are most sensitive to stress. Another model can be based on cannabis or other substances; one may expect escalating substance use to be the major factor for relapse induction and thus select NLP variables with maximum sensitivity to the dose of recreational drugs. Our results should not be taken to indicate the superiority of disorganization/FTD-based theory to these other alternatives, which have yet to be tested.

Our study has several strengths as well as limitations. The recruitment of initially untreated individuals for speech assessment, along with monthly follow-up and medical record tracking for outcome evaluation, enabled prospective model-building for early relapse. Nevertheless, we had only 12 relapses out of 68, greatly limiting the ability to test more predictors. In large-scale studies with more events of interest, a partitioned sub-sample could provide out-of-sample construct identification and variable selection of speech-NLP markers. Our dependence on a single speech assessment and lack of measurement of events that immediately preceded a relapse (e.g. discontinuation, escalation in cannabis use) limited the model to one that can be employed only at the time of first clinical presentation. Further, our aim to recruit consenting individuals with untreated first episode introduced a selection bias. This excluded involuntary patients, especially in forensic settings who were incapable of consenting. These issues may explain lower relapse rates reported here (18 % of patients in the first year). It is important to note that relapses tend to recur in the same individuals; our analysis did not consider relapse *events* per se as the unit of interest but focused on those who relapsed (i.e., *individuals* as the unit of interest, rather than the number of relapses' experienced by an individual). As ‘relapse begets relapse’, but the relapses per se do not seem to affect the severity of subsequent positive FTD burden (unlike the effects seen on delusions in a 10-year follow-up study; Hui et al., 2018), we can anticipate the language variables studied here to have comparable predictive value for subsequent episodes. We also took a single speaker's production perspective in the linguistic analysis, which misses the valuable dialogical information (Olarewaju et al., 2023) and deficits in pragmatics (Agostoni et al., 2021; Pawełczyk et al., 2021; Perlini et al., 2012) relevant for psychosis. Nevertheless, showing a picture and recording what patients say is highly feasible relative to many other approaches to the study of communication in clinical practice (Brederoo et al., 2021). Although this approach may decrease ecological validity, it provides a practical approach to implementation of these methods in a clinical setting. Finally, we operationalized relapse as hospitalization; we did not have data on other help-seeking events (e.g., more frequent visits to see a clinical case manager or medication increase) or changes in symptom severity on clinical scales. While this approach has high specificity (all counted relapses are likely to be true psychotic relapses (Addington et al., 2013), sensitivity is likely to be low, and the true frequency of relapse events is likely to be higher than the 18 % of individuals with early relapse that we report here.

To conclude, our study provides the proof of concept for theoretically motivated variable selection for risk prediction approaches in psychosis. By employing a Bayesian approach for clinical knowledge-driven feature search, we can reduce the dimensionality and improve the interpretability of quantitative models, both of which are necessary to transfer predictive insights to the clinic. The resulting sparse models are likely to perform better than clinical profiles currently used for prognostication.

## CRediT authorship contribution statement

**Tyler C. Dalal:** Writing – review & editing, Writing – original draft, Methodology, Investigation, Formal analysis, Conceptualization. **Min Tae M. Park:** Writing – review & editing, Writing – original draft, Formal analysis, Data curation, Conceptualization. **Angelica M. Silva:** Writing – review & editing, Writing – original draft. **Svetlana Iskhakova:** Writing – review & editing, Resources. **Alban Voppel:** Writing – review & editing. **Noah J. Brierley:** Writing – review & editing. **Michael MacKinley:** Writing – review & editing, Data curation. **Emmanuel Olarewaju:** Writing – review & editing. **Lena Palaniyappan:** Writing – review & editing, Writing – original draft, Supervision, Methodology, Investigation, Conceptualization.

## Ethics statement

The studies involving human participants were reviewed and approved by the Western University Health Sciences Research Ethics Board. The patients/participants provided written informed consent to participate in this study.

## Funding

The TOPSY study was funded by the 10.13039/501100000024Canadian Institutes of Health Research (CIHR) Foundation Grant (Grant no. 375104/2017) to LP with data acquisition supported by the Canada First Excellence Research Fund to BrainSCAN, Western University (Imaging Core); Innovation fund for Academic Medical Organization of Southwest Ontario (PROSPECT study). Compute Canada Resources (Application No. 1530) were used in the storage and analysis of imaging data. LP acknowledges research support from the Canada First Research Excellence Fund, awarded to the Healthy Brains, Healthy Lives initiative at McGill University (through New Investigator Supplement to LP); Monique H. Bourgeois Chair and Graham Boeckh Foundation (McGill University) and salary award from the Fonds de recherche du Québec-Santé (FRQS #366934). TCD was supported by Chrysalis Foundation (London Ontario), AMS's position was funded by a Project Grant from the Canadian Institutes of Health Research (Grant no. FRN 391348). AMS received John. R Hayes Award for Excellence in Writing Research. AV is supported by a Wellcome Discretionary Grant (Palaniyappan/Sommer: 226168/Z/22). EO received funding from the Healthy Brain, Healthy Lives (HBHL) Fellowship and Graduate Excellence Recruitment award from McGill University.

## Declaration of competing interest

LP reports personal fees for serving as chief editor from the Canadian Medical Association Journals, speaker/consultant fee from Janssen Canada and Otsuka Canada, SPMM Course Limited, UK, Canadian Psychiatric Association; book royalties from Oxford University Press; investigator-initiated educational grants from Janssen Canada, Sunovion and Otsuka Canada outside the submitted work. All other authors report no potential conflicts.

## Data Availability

The authors have made the anonymized transcripts of the data used in the study available through the DISCOURSE consortium's Psychosis Talkbank https://psychosis.talkbank.org/ as per the Institutional Ethics stipulations. Requests to access the datasets should follow the instructions on this website. DISCOURSE consortium membership is open for all qualified researchers through https://discourseinpsychosis.org/.

## Appendix A Supplementary data

Supplementary material Image 1
