# Supplementary material for: Clinical psychopathology-based early relapse prediction model using speech and language in psychosis
Source: Schizophr Res Cogn. 2025 Sep 23;43:100392. doi: 10.1016/j.scog.2025.100392 (PMC12493218; doi:10.1016/j.scog.2025.100392)
Supplement: Supplementary file 1 — Supplementary material [file mmc1.docx]

**Participant recruitment**

Recruitment was carried out between April 2017 and December 2022. Patients with FEP were recruited from the Prevention and Early Intervention for Psychosis Program (PEPP) in London, Ontario, Canada, upon referral following their first presentation of psychosis. Presence of criterion A features of DSM-5 schizophrenia criteria (i.e. a psychotic episode) was necessary for inclusion; patients who had a history of significant medical illness contributing to psychotic symptoms, known intellectual/developmental disability precluding assessment of language, and those who had longer than 2 weeks of lifetime antipsychotic exposure, were excluded. Substance use (cannabis, nicotine and alcohol) was not an exclusion criterion; however, psychosis associated with states of intoxication was excluded, and screening to assess the frequency of reported substances was completed upon referral. FEP subjects were assessed within their first week of referral by the FEP team and as such, had <2 weeks of lifetime antipsychotic exposure.

Diagnosis was confirmed 6-12 months after first contact, based on the best estimate procedure (Leckman et al., 1982) based on all available information at that stage, including the Structured Clinical Interview for DSM-5 of the American Psychiatric Association (Association, 2013). A minimum of 3 members of the clinical and research team (2 research psychiatrists and the care provider from the PEPP clinic) participated in the consensus process to determine diagnosis at follow-up (see Table 1 for follow-up diagnosis). Of the 73 recruited, 1 withdrew consent for further data use, 3 did not have speech data of satisfactory quality for further use, and 1 did not have traceable clinical records to determine relapse outcome with certainty. The final sample included N=68 individuals, with 18% relapsing in 1 year (n=12).

**Picture description task**

Subjects described the characteristics of the three pictures originally from the Thematic Apperception Test (Murray, 1943), one after the other for 1 minute each, as part of the Thought and Language Index administration procedures (TLI). Interviewers provided minimal prompting only to ensure capturing enough speech in the subject's response. Responses (including interviewer’s prompts, if any) were recorded and then transcribed to text, with only the patient’s transcribed speech retained for analysis.

Picture descriptions from the TLI elicitation procedures were recorded and transcribed manually to plain text. Punctuations (other than sentence dividers) were removed while any repetitions (e.g., “..because.. because…”) and non-fluencies (e.g., fillers) were preserved as in previous analyses to maintain the natural context of oral speech productions. This was done by 2 trained graduate research assistants to ensure reliability between raters and consistency among speech samples. This process reduced the variations in sentence splitting that may result from subsequent automated language processing.

**Relapse assessment**

Case notes were studied using a checklist to determine relapse within 1 year after reaching clinical remission (CGI<3), estimated based on medical, social work, occupational therapy and nursing notes during the period of follow-up. Specifically, details of all emergency department visits, direct inpatient psychiatric admissions, and all outpatient visits were reviewed for 1 year after treatment stabilization in the PEPP program. For emergency department visits, we documented reasons for visit (i.e. whether related to mental health or not), symptoms reported and whether a psychiatric consultation was requested. We also reviewed whether patients were directly admitted from an outpatient appointment. In our sample, a psychotic relapse identified at the emergency department always led to an inpatient admission, i.e., there were no ED visits with psychotic symptoms in the 1st year that were turned away from admission. Thus, we had n=7 psychiatric admissions via emergency visits and n=5 other admissions directly from the outpatient unit (PEPP clinic) following a change in clinical status (CGI>3 with psychotic symptoms). Therefore, an inpatient admission within one year always indicated a psychotic relapse after initial improvement, with n=12 meeting these criteria.

**Language markers**

The language data for each participant was analyzed through three language processors (Figure 2) to determine individual scores.

The Analytic Thinking Index derived from the LIWC-22 (Boyd et al., 2022). This language processor is based on the count of Function words (articles, prepositions, personal pronouns, impersonal pronouns, auxiliary verbs, adverbs, conjunctions, and negations). We adjusted this index for verbosity by dividing the analytic-thinking-index (ATI) by number of spoken words during the task.

Clause Complexity measure refers to the relationships between embedded clauses in a sentence. The more clauses constituting the syntactic structure, the higher complexity of syntax is produced (See (Hawkins, 1994). This metric was obtained using the Tool for Automatic Analysis of Syntactic Sophistication and Complexity (TAASSC 1.3.8; Kyle and Crossley, 2018) which scored 31 granular indices summed as the value of clause complexity.

Semantic similarity score was calculated as the average cosine similarity between progressive adjacent sentence pairs using the bag-of-words approach (i.e. word order is disregarded with root sum square of vector weights for each token calculated). Finally, provided by the Tool for the Automatic Analysis of Cohesion (TAACO 2.0.4; Crossley et al., 2019), we used both word2vec and latent Semantic Analysis approaches and used the average of the two as the unique semantic similarity score. While prediction-based word2vec appears to provide better embeddings (see Just et al., 2023), count-based LSA performs better with scarce data, as in shorter speech transcripts (Altszyler et al., 2017). In this work, all language processors incorporated a part-of-speech (POS) tagger from the Natural Language Toolkit, and synonym sets from the WordNet lexical database, which reflect a corpus-based approach for quantitative linguistic analysis.

Altszyler, E., Sigman, M., Ribeiro, S., Slezak, D.F., 2017. Comparative study of LSA vs Word2vec embeddings in small corpora: a case study in dreams database. Consciousness and Cognition 56, 178–187. https://doi.org/10.1016/j.concog.2017.09.004

Association, A.P., 2013. Diagnostic and Statistical Manual of Mental Disorders (DSM-5®). American Psychiatric Pub.

Boyd, R.L., Ashokkumar, A., Seraj, S., Pennebaker, J.W., 2022. The development and psychometric properties of LIWC-22. Austin, TX: University of Texas at Austin 1–47.

Crossley, S.A., Kyle, K., Dascalu, M., 2019. The Tool for the Automatic Analysis of Cohesion 2.0: Integrating semantic similarity and text overlap. Behavior Research Methods 51, 14–27. https://doi.org/10.3758/s13428-018-1142-4

Hawkins, J.A., 1994. A performance theory of order and constituency, A performance theory of order and constituency. Cambridge University Press, New York, NY, US.

Just, S.A., Bröcker, A.-L., Ryazanskaya, G., Nenchev, I., Schneider, M., Bermpohl, F., Heinz, A., Montag, C., 2023. Validation of natural language processing methods capturing semantic incoherence in the speech of patients with non-affective psychosis. Front. Psychiatry 14. https://doi.org/10.3389/fpsyt.2023.1208856

Kyle, K., Crossley, S.A., 2018. Measuring Syntactic Complexity in L2 Writing Using Fine-Grained Clausal and Phrasal Indices. The Modern Language Journal 102, 333–349. https://doi.org/10.1111/modl.12468

Leckman, J.F., Sholomskas, D., Thompson, D., Belanger, A., Weissman, M.M., 1982. Best Estimate of Lifetime Psychiatric Diagnosis: A Methodological Study. Arch Gen Psychiatry 39, 879–883. https://doi.org/10.1001/archpsyc.1982.04290080001001

Murray, A., 1943. Thematic apperception test. Harvard University Press, Cambridge, MA, US.
